# Insights into *bs5* resistance mechanisms in pepper against *Xanthomonas euvesicatoria* through transcriptome profiling

**DOI:** 10.1186/s12864-024-10604-8

**Published:** 2024-07-23

**Authors:** Aastha Subedi, Gerald V. Minsavage, Pamela D. Roberts, Erica M. Goss, Anuj Sharma, Jeffrey B. Jones

**Affiliations:** 1 Department of Plant Pathology, University of Florida, Gainesville, FL USA; 2 Southwest Florida Research & Education Center, University of Florida, Immokalee, FL USA; 3 Emerging Pathogens Institute, University of Florida, Gainesville, FL USA; 4 Department of Horticultural Sciences, Gulf Coast Research and Education Center, University of Florida, Wimauma, FL USA

**Keywords:** Transcriptomics, *bs5*, ECW, *Xanthomonas*, Bacterial-spot, PTI, Recessive-resistance

## Abstract

**Background:**

Bacterial spot of pepper (BSP), caused by four different *Xanthomonas* species, primarily *X. euvesicatoria (Xe)*, poses a significant challenge in pepper cultivation. Host resistance is considered the most important approach for BSP control, offering long-term protection and sustainability. While breeding for resistance to BSP for many years focused on dominant R genes, introgression of recessive resistance has been a more recent focus of breeding programs. The molecular interactions underlying recessive resistance remain poorly understood.

**Results:**

In this study, transcriptomic analyses were performed to elucidate defense responses triggered by *Xe* race P6 infection by two distinct pepper lines: the *Xe*-resistant line ECW50R containing *bs5*, a recessive resistance gene that confers resistance to all pepper *Xe* races, and the *Xe*-susceptible line ECW. The results revealed a total of 3357 upregulated and 4091 downregulated genes at 0, 1, 2, and 4 days post-inoculation (dpi), with the highest number of differentially expressed genes (DEGs) observed at 2 dpi. Pathway analysis highlighted DEGs in key pathways such as plant-pathogen interaction, MAPK signaling pathway, plant hormone signal transduction, and photosynthesis – antenna proteins, along with cysteine and methionine metabolism. Notably, upregulation of genes associated with PAMP-Triggered Immunity (PTI) was observed, including components like FLS2, Ca-dependent pathways, Rboh, and reactive oxygen species (ROS) generation. In support of these results, infiltration of ECW50R leaves with bacterial suspension of *Xe* led to observable hydrogen peroxide accumulation without a rapid increase in electrolyte leakage, suggestive of the absence of Effector-Triggered Immunity (ETI). Furthermore, the study confirmed that *bs5* does not disrupt the effector delivery system, as evidenced by incompatible interactions between avirulence genes and their corresponding dominant resistant genes in the *bs5* background.

**Conclusion:**

Overall, these findings provide insights into the molecular mechanisms underlying *bs5*-mediated resistance in pepper against *Xe* and suggest a robust defense mechanism in ECW50R, primarily mediated through PTI. Given that *bs5* provides early strong response for resistance, combining this resistance with other dominant resistance genes will enhance the durability of resistance to BSP.

**Supplementary Information:**

The online version contains supplementary material available at 10.1186/s12864-024-10604-8.

## Introduction

Pepper (*Capsicum annum L*.) plays a vital role in global agriculture, holding substantial economic significance as one of the most versatile and valuable vegetable crops worldwide. As of 2022, the global pepper production reached around 3.6 million tons, with China, India, and Mexico reporting highest production [1]. Despite its importance, pepper growers face constant threats, particularly from diseases like bacterial spot, leading to significant yield losses. Bacterial spot of pepper (BSP) is caused by four different *Xanthomonas* species, although *X. euvesicatoria (Xe)* is primarily responsible for this disease worldwide [2, 3]. Despite deploying control methods like using copper-based bactericides, challenges continue due to the emergence of copper resistance in *Xanthomonas* populations [4–6].

Host resistance is considered the most important method to control bacterial spot disease due to its inherent ability to provide long-term protection and serve as a sustainable solution. Plants exhibit two types of innate immune system, with the first layer activating PAMP-Triggered Immunity (PTI) upon detecting pathogen-associated molecules [7]. Despite pathogens deploying virulence molecules or proteins to disrupt host immunity, plants employ Effector-Triggered Immunity (ETI), as a second robust immune signaling pathway triggered by the recognition of effectors by NLRs [7]. Pepper plants have also evolved effective mechanisms to recognize and respond to BSP through resistance genes. Five dominant hypersensitive resistance (R) genes (*Bs1*,* Bs2*,* Bs3*,* Bs4*,* Bs7*) have been identified in pepper, with *Bs1*,* Bs2*, and *Bs3* being successfully incorporated into commercial pepper cultivars [8, 9]. However, these dominant R genes are ineffective when avirulence genes are absent, as observed in pepper race 6 (P6), rendering it virulent on commercial pepper cultivars. The survey of Southwest Florida pepper fields from 2019 to 2021 revealed predominant races P1 (42%), P6 (26%), and P3 (24%), alongside less common P4 (8%), potentially limiting the efficacy of commercialized dominant R genes due to the prevalence of P6 [3]. Two recessive genes designated as *bs5* and *bs6*, were found to be resistant to all pepper races including P6 and resistance was not associated with any of the known avirulence (effector) genes [10]. Characterization of these two recessive genes revealed that *bs5* confers a greater level of resistance than *bs6*, however, in combination they confer full resistance to P6 at higher temperatures [11]. Furthermore, *bs5* has been incorporated into commercial varieties [12, 13]. Another recessive gene *bs8* was also identified in *C. annuum* accession PI 163192 conferring resistance against *X. gardneri* [14].

Recessive resistance, a less explored form of resistance in comparison to dominant traits, is not limited to specific pathogenic races, particularly in the tomato/pepper- *Xanthomonas* pathosystem, and usually arises from the alteration or loss of host susceptibility (S) factors [15]. Despite challenges in breeding, recessive resistance is valuable for addressing rapidly evolving bacterial pathogens such as *Xe*, offering potential for durable protection. The *bs5* gene was first reported as a monogenic, recessive, non-hypersensitive resistance against all races of *Xe* [10], and was found to have originated from *Capsicum annuum* PI 271322 [16]. Jones et al. [10] employed repeated backcrosses to introgress *bs5* into the pepper Early Calwonder (ECW) background, resulting in the development of the ECW50R line [11]. The *bs5* resistant allele was identified to encode a Cysteine-Rich Transmembrane Module (CYSTM) protein that is 2 amino acids shorter in length than the wild-type Bs5 protein [17, 18].

RNA-sequencing is a useful technique for monitoring transcriptional changes in cells over time to better understand host-pathogen interactions [19], identifying biomarkers [20], and discovering potential therapeutic targets in various organisms [21]. In pepper, RNA-sequencing has been used to understand underlying genes and signaling pathways involved in the resistance network under biotic [22] and abiotic stresses [23]. Transcriptomic analyses have been extensively used to explore response to bacterial spot in tomato [24–27], however, limited studies have been reported for pepper [28] and only one genome-wide comprehensive analysis of BSP infection – to our knowledge – with dominant resistance in pepper has recently been completed [29]. A deeper understanding of the responses involved in defense signaling pathways to BSP, particularly related to recessive resistance, can provide insights into the molecular mechanisms underlying disease resistance.

This study provides a comprehensive exploration of the transcriptional changes between a pepper line with recessive resistance (*bs5*) and a susceptible cultivar (ECW) in response to infection with a P6 strain of *Xe*. By studying the transcriptomic aspects of these contrasting responses, our goal is to explore the molecular mechanisms governing the interaction between peppers and *Xe* and determine key pathways linked to disease resistance. To further understand whether *bs5* induces rapid cell death and to assess the impact of the *bs5* gene on the *in planta* effector delivery system, an evaluation of electrolyte leakage and the hypersensitive response reaction was also performed. This work enhances our basic understanding of recessive resistance in pepper and opens the door to the development of more resilient and disease-resistant pepper cultivars.

## Methodology

### Pathogen inoculation and sample collection

*C. annuum* cv. Early Calwonder (ECW) is a bacterial spot-susceptible bell pepper cultivar (first commercialized in 1940’s) and used as recurrent parent for bacterial spot resistance breeding at the University of Florida. ECW50R is a near-isogenic line of ECW (developed at the University of Florida) that carries the *bs5* resistance introgression from pepper accession PI271322 [10, 17] and is resistant to all races of *Xe*. Both lines were cultivated under standard greenhouse conditions. A P6 *Xe* strain 21_52_F1 [3] isolated from pepper from Florida in 2021 was used for infiltration. Bacterial cells from an overnight culture grown on Nutrient Agar at 28˚C, were suspended in sterile tap water and optically adjusted to a concentration of 10^8^ CFU/mL (OD_600_ = 0.3). The resulting suspension was diluted to a final concentration of 10^6^ CFU/mL, which was infiltrated into the leaf apoplast of 6-week-old plants using a sterile syringe. The mock inoculation treatment consisted of infiltrating with sterile tap water. Twelve plants of each genotype (ECW and ECW50R) were inoculated with bacterial suspension, and tissue samples from inoculated areas were harvested at 0 (~ 30 min post inoculation), 1, 2 and 4 days post inoculation (dpi) from *Xe*-inoculated leaves, denoted as ECW_0, ECW_1, ECW_2, ECW_4, ECW50R_0, ECW50R_1, ECW50R_2, ECW50R_4, respectively. For the mock inoculation (water), three plants of each genotype were infiltrated, and samples were collected at 0 dpi, labeled as ECW_Wa and ECW50R_Wa, respectively. Three independent biological replicates were used for each treatment, resulting in a total of 30 samples [3 biological replicates × (4 timepoints for bacteria inoculation + 1 for mock inoculation with water) × 2 genotypes]. The collected samples were promptly snap-frozen in liquid nitrogen, ground with Qiagen TissueLyser II, and stored at − 80˚C for subsequent RNA extraction and analysis.

### RNA extraction and transcriptome sequencing

Total RNA was extracted using RNeasy Plant Mini Kit (Qiagen GmbH, Hilden, Germany) according to the manufacturer’s instructions. The quality and quantity of RNA was assessed using NanoDrop (Thermo Fisher Scientific, USA) before sending to SeqCenter LLC (Pittsburgh, PA, USA) for sequencing. Library preparation was performed using Illumina Stranded Total RNA Prep with Ribo-Zero Plus kit (Illumina Inc, San Diego, CA) and 10 bp unique dual indices (UDI). Briefly, strand information is captured using deoxyuridine triphosphates (dUTPs) in the second strand synthesis step of cDNA synthesis. After adapter ligation, second strand amplification was suppressed in the final library amplification due to polymerase stalling at the location of the incorporated dUTPs. Sequencing was done using Illumina NovaSeq X Plus (Illumina Inc, San Diego, CA), producing 2 × 151 bp paired-end reads. Demultiplexing, quality control, and adapter trimming was performed with bcl-convert (v4.1.5). The RNA sequencing raw data has been deposited into the NCBI SRA database with the BioProject number: PRJNA1099765 and their BioSample accessions are listed in Table S1.

### Transcriptome analysis pipeline

Sequence analysis was performed on the University of Florida HiPerGator supercomputer platform using the publicly available pipeline at github.com/rknx/RNAseq [30]. Initially, sequence quality was assessed through FastQC (bioinformatics.babraham.ac.uk/projects/fastqc). Subsequently, reads were aligned to the reference genome of pepper cultivar Zunla-1 (NCBI Accession GCA_000710875.1) [31] using HiSAT2 with default settings [32]. The resulting files were then compressed into binary format using SAMTools [33]. Transcript abundance was quantified using HTSeq2 [34].

### Differential Gene expression (DGE) analysis

Differentially expressed genes were identified using the DESeq2 R package [35] using the raw counts from HTSeq2 as inputs. The expression levels of the inoculated samples from all four timepoints were compared to those of mock inoculation (water) for ECW and ECW50R separately. In addition, the expression of genes were also compared across the two genotypes at all timepoints. The significance of the relative change in expression, i.e., log 2-fold change (log2FC), was assessed for each gene using Wald’s test, then subsequently adjusted through the Benjamini–Hochberg method to derive the adjusted p-value (p.adj) [36]. Genes with an absolute log2FC ≥ 1 and p.adj ≤ 0.05 were considered significantly differentially expressed.

### KEGG enrichment analysis

To elucidate the potential functions and pathways associated with identified Differentially Expressed Genes (DEGs), Kyoto Encyclopedia of Genes and Genomes (KEGG) pathway analysis was performed [37] using the ClusterProfiler package [38] in R. Enrichment analyses were conducted comparing the two genotypes for each timepoint, considering all differentially expressed genes, as well as separately for upregulated and downregulated genes. The magnitude and significance of expression changes for genes in key pathways were visualized using the PathView package [39].

### Generation of *fliC* Deletion Mutants

Nucleotide sequences of *Xe* strain 21_52_F1 flanking *fliC* gene were identical to those in *X. fuscans* subsp. *aurantifolii* strain *ICPB 10535*, hence a suicide cloning vector designed to disrupt *fliC* in latter was used for this experiment. To generate this vector, *fliC* gene from *ICPB 10535* strain was amplified (forward primer (FP) FLY-F: AGTCACCCTCAAGACCAGCC and reverse primer (RP) FLY-R: CGCTGCTGATCACCTTGTCC) using GoTaq DNA polymerase (Promega, Madison, WI) in a Mycycler thermal cycler (Bio-Rad, Hercules, CA). The amplicon was purified and cloned into pGEM-T Easy vector (Promega, Madison, WI). Outwards facing primers (FP DELFLY-F: CCC*GGATCC*ACCATCGCCAACCTGAGCG and RP DELFLY-R: CCC*GGATCC*AGACGACAGACGCTGGATGC) with hanging *Bam*HI restriction sites were used to generate linear vector missing internal region of *fliC*. The ends of this vector were digested with *Bam*HI and recircularized. The *fliC* fragment with internal deletion (ΔfliC) was cut out from pGEM-T using *Apa*I and *Spe*I, inserted into the suicide plasmid, pOK1, digested with *Apa*I and *Xba*I, and transformed into *Escherichia coli* - λpir. pOK1:ΔfliC was transferred into P6 strain *Xe* 21_52_F1 by homologous recombination with helper plasmid, pRK2013 as described previously [40]. The correct deletion of entire *fliC* (including flg22 region - an epitope of *fliC* recognized by plant FLS2 [41, 42]) in *Xe* 21_52_F1 was confirmed through PCR (FP fli_Up: GTGACGCTCTGATCGCCATA and RP fli_Down: TGGAAGTTACAGCGTGCAGT) and additionally by a motility test in 1% tryptone and 0.4% Agar.

### In Planta Population Growth Experiment

To determine if flg22 triggers *bs5*-mediated defense responses through FLS2 mediated pathways, wildtype *Xe* 21_52_F1 strain and its *fliC* mutant were infiltrated into leaves of ECW and ECW50R genotypes at concentration of 10^5^ CFU/mL. Leaf samples from infiltrated area were collected at 0, 3, 6, and 9 days after inoculation (dpi). Leaf disks from the inoculated region were macerated in sterile tap water. Ten-fold serial dilutions of the macerate were then plated on Nutrient Agar and incubated at 28 °C for 72 h. The *in planta* bacterial populations were estimated from the number of colonies in the agar plates and the dilution ratio. Paired sample t-test was performed to determine the significant differences between two populations.

### Histochemical assays using DAB staining

The staining procedure for 3,3’-diaminobenzidine (DAB) was performed as previously described [43]. A 10^8^ CFU/mL suspension of *Xe* strain 21_52_F1 was infiltrated into the leaves ECW and ECW50R plants. Leaf samples were collected at 0, 1, 2, and 4 days post-inoculation (dpi) for DAB staining. Leaves were washed three times with double distilled (dd) H_2_O and incubated overnight with 1 mg/mL of DAB in staining solution (50 mM boric acid buffer, pH 7.6). The samples were then de-stained by boiling in ethanol.

### Determination of electrolyte leakage from infiltrated leaf tissue

Tissue necrosis resulting from the infiltration of bacterial suspension into leaves was evaluated by measuring electrolyte leakage by following previously published method [44]. Leaves of both ECW50R and ECW lines were infiltrated with suspension of *Xe* strain 21_52_F1 adjusted to 10^8^ CFU/mL (OD_600_ = 0.3). The inoculated plants were maintained in a greenhouse with a 12-hour light period at 26 °C and a 12-hour dark period at 16 °C. Every 12 h, starting from the initial 0-hour mark, three leaves per genotype were collected and independently assessed for electrolyte leakage. Electrolyte leakage was quantified by measuring conductivity at 28°C. A paired sample t-test was conducted to identify significant differences in electrolyte leakage between two genotypes.

### Plant inoculations to test for effector delivery system

To assess the impact of *bs5* gene on *in planta* effector delivery system, four *Xe* pathogen races (P3, P4, P5, and P6) were infiltrated into ECW12346, which carries dominant resistant genes (*Bs1*,* Bs2*, and *Bs3*) and recessive resistance genes (*bs5* and *bs6*). Additionally, three pepper genotypes (ECW10R, ECW20R, and ECW30R), each carrying a single resistance gene (*Bs1*,* Bs2*, and *Bs3*, respectively), were also infiltrated. Positive controls (P5: 82 − 8 UNS::pXvCu, P3: 88 − 5, and P4: 95 − 2) for incompatible reactions with *Bs1*,* Bs2*, and *Bs3*, respectively, were used, while P6 *Xe* 21_52_F1 served as a negative control for an incompatible reaction [3, 8]. Bacterial suspensions adjusted to 10^8^ CFU/mL (OD_600_ = 0.3) from overnight cultures were infiltrated into leaves, and hypersensitive responses (HR) or susceptible reactions were monitored at 12, 24, 36, 48 and, 72 h post-inoculation (hpi).

## Results

### Transcriptomics assembly statistics

A total of 157 billion base pairs of sequence was generated across all samples using an Illumina NovaSeq X Plus sequencing platform, resulting in around 5.2 Gbp sequences per sample (Supplementary Table S1). On average, 85.56% of the reads were successfully mapped to the pepper reference genome Zunla-1 (Supplementary Table S1). A total of 27,826 genes were identified. Within specific samples (ECW_Wa, ECW_0, ECW_1, ECW_2, ECW_4, ECW50R_Wa, ECW50R_0, ECW50R_1, ECW50R_2, ECW50R_4), the expressed genes numbered 25,052, 24,736, 24,790, 24,801, 24,524, 25,092, 24,840, 24,637, 24,516, and 24,265, respectively (Supplementary Table S1).

### Differentially expressed genes (DEGs) in ECW50R and ECW inoculated leaves at different timepoints

**Fig. 1:**
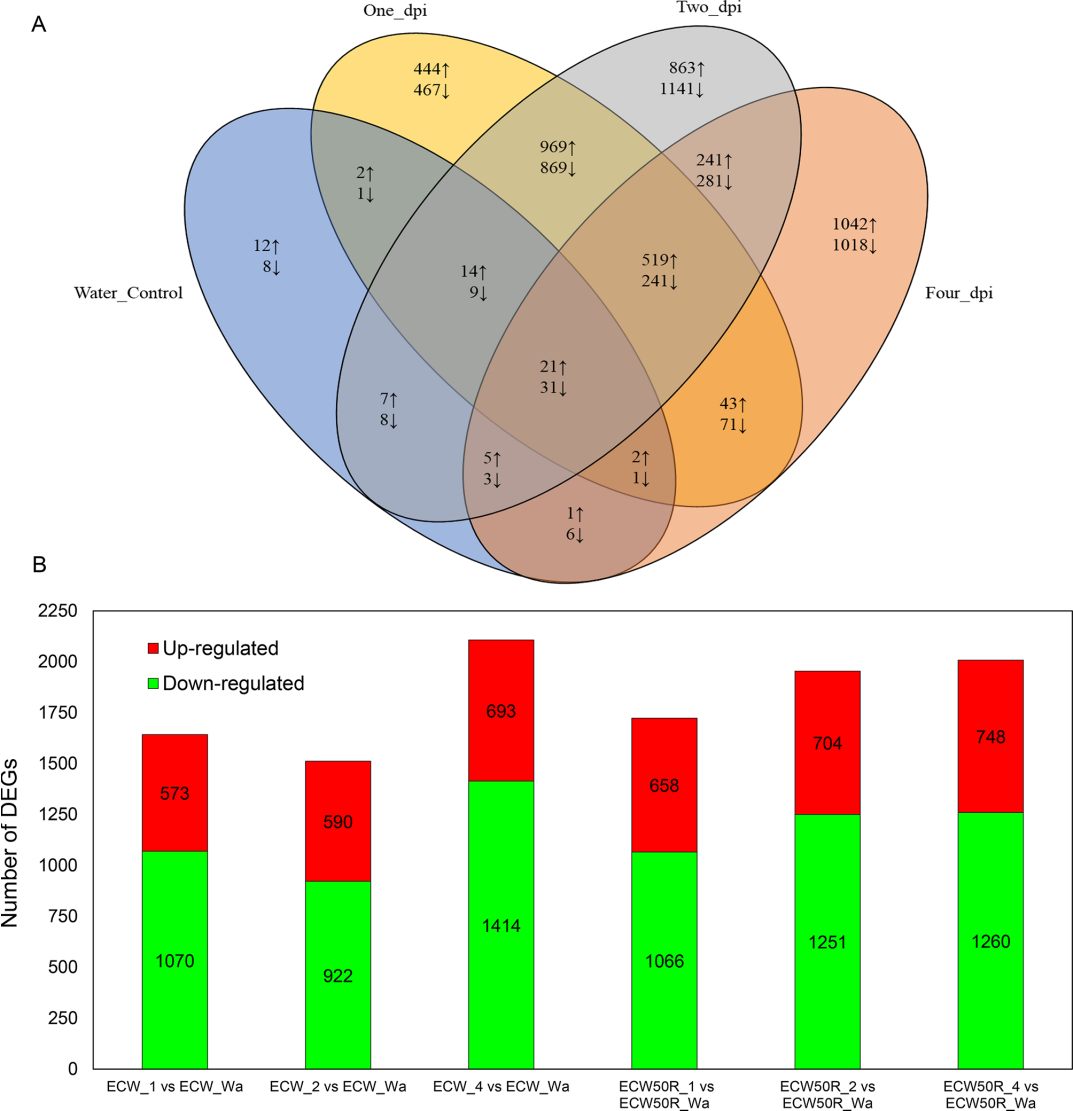
(**A**) Number of differentially expressed genes (DEGs) in ECW50R line in comparison to ECW in response to *X. euvesicatoria* 21_52_F1 (P6) infection at different timepoints. ↑ represents number of upregulated DEGs (|log2(fold change) | ≥ 1) in ECW50R line in comparison to ECW. ↓ represents number of downregulated DEGs (|log2(fold change) | ≤ -1) in ECW50R line in comparison to ECW. (**B**) Number of up and down regulated DEGs at 1, 2 and 4 dpi in ECW50R and ECW with respect to water control for each genotype

For all comparisons, genes with less than 2-fold change in expression (|log2fc < 1|) and adjusted p-value greater than 0.05 (p.adj > 0.05) were considered not significantly differentially expressed and subsequently filtered out. A comparative analysis of DEGs in sample pairs of ECW_0 vs. ECW_Wa and ECW50R_0 Vs ECW50R_Wa showed no significantly differentially expressed genes between mock-inoculated (water) and 0 dpi inoculation, hence further analysis was carried out using pepper leaves of respective lines with water as control samples. DGE analysis was also performed between the resistant and susceptible genotypes at each sampling time (ECW50R_Wa vs. ECW_Wa, ECW50R_1 vs. ECW_1, ECW50R_2 vs. ECW_2, ECW50R_4 vs. ECW_4) to reveal the expression patterns of genes in ECW50R that may play a role in resistance to *Xe*. DESeq2 identified 131 (64 up and 67 down-regulated), 3704 (2014 up and 1690 down-regulated), 5222 (2639 up and 2583 down-regulated) and 3526 (1874 up and 1652 down-regulated) DEGs in ECW50R relative to ECW between water controls and at 1, 2 and 4 dpi, respectively (Fig. 1A). When a more stringent measure of |log2fc|<8 was used, a total of 59 DEGs were identified, most of which were downregulated at 4 dpi in ECW50R relative to ECW, while the upregulated genes were annotated as involved in disease resistance (Supplementary Table S2).

To investigate the expression patterns of genes in ECW50R and ECW individually during the different timepoints after *Xe* infection, gene expression was also compared across different timepoints for each genotype i.e., ECW_1 vs. ECW_Wa, ECW_2 vs. ECW_Wa, ECW_4 vs. ECW_Wa, ECW50R_1 vs. ECW50R_Wa, ECW50R_2 vs. ECW50R_Wa, ECW50R_4 vs. ECW50R_Wa. DESeq2 identified 1643 (573 upregulated and 1070 down regulated), 1512 (590 upregulated and 922 down regulated), and 2107 (693 upregulated and 1414 down regulated) DEGs at 1, 2 and 4 dpi in ECW, respectively, compared to the water control. Similarly, 1724 (658 upregulated and 1066 down regulated), 1955 (704 upregulated and 1251 down regulated), and 2008 (748 upregulated and 1260 down regulated) DEGs were identified at 1, 2 and 4 dpi in ECW50R line, respectively, compared to the water control (Fig. 1B). Overall, both the resistant genotype, ECW50R, and the susceptible genotype ECW had the highest number of DEGs at 4 dpi, compared to water control (Fig. 1B) suggesting that the timing of the host plant response to *X. euvesicatoria* infection is similar between the two genotypes.

### Significantly enriched pathways

**Fig. 2:**
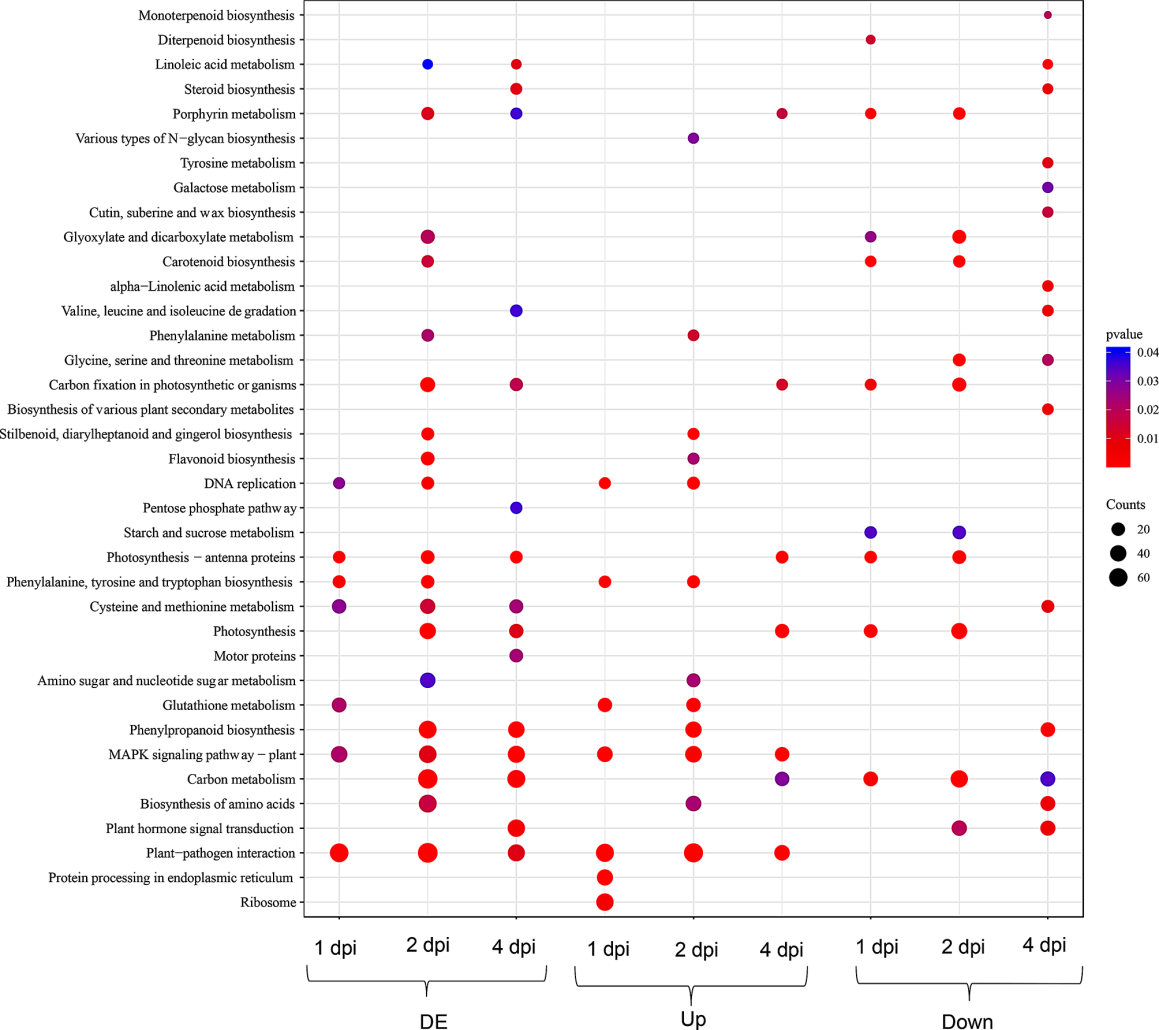
Significantly enriched (Differentially Expressed- DE, Upregulated-Up and Downregulated-Down) KEGG pathways in ECW50R relative to ECW at 1, 2 and 4 dpi

From the KEGG enrichment analysis, “Plant Pathogen Interaction (PPI)”, “Photosynthesis – antenna proteins”, “Cysteine and methionine metabolism”, and “MAPK signaling pathway − plant” were the most common significantly enriched pathways in ECW50R compared to ECW across different timepoints (1,2,4 dpi) (Fig. 2). At 2 dpi, a lot of pathways were found to be enriched, with PPI having the lowest p-value (Fig. 2). At 4 dpi, most of the pathways observed at 2 dpi were still enriched, however the number of significantly enriched pathways were less than at 2 dpi (Fig. 2). PPI and MAPK signaling pathways were upregulated in ECW50R compared to ECW at all three timepoints (Fig. 2). Photosynthesis related pathways were downregulated at 1 and 2 dpi and upregulated at 4 dpi in ECW50R compared to ECW. Plant hormone signal transduction was downregulated at 2 and 4 dpi in ECW50R compared to ECW. The phenylpropanoid biosynthesis, steroid biosynthesis, monoterpenoid biosynthesis, biosynthesis of various secondary metabolites and amino acids were all downregulated at 4 dpi (Fig. 2) in ECW50R compared to ECW.

In ECW50R, pathways related to DNA replication, ribosome and amino acid synthesis, glutathione metabolism were upregulated in 1 dpi compared to ECW50R_Wa, but more pathways were downregulated than upregulated (Fig. S1). MAPK signaling pathway and phenylpropanoid biosynthesis were upregulated at 2 and 4 dpi compared to water control. PPI pathway was found to be enriched among significantly upregulated genes at 2 and 4 dpi as well as significantly downregulated genes at 1 dpi and 4 dpi. Photosynthesis related and plant hormone signal transduction were downregulated at 1, 2 and 4 dpi relative to ECW50R_Wa (Fig. S1).

In comparison across timepoints in ECW genotype, PPI was downregulated at all timepoints in ECW compared to ECW_Wa whereas MAPK- signaling pathway was significantly upregulated as well as downregulated at both 1 and 2 dpi compared to ECW_Wa, but not at 4 dpi (Fig. S2). Plant hormone signal transduction was downregulated at 1 dpi and 4 dpi, however, upregulated at 2 dpi compared to water control. Photosynthesis related genes were downregulated at 4 dpi only compared to ECW_Wa (Fig. S2).

### Expression of genes involved in plant pathogen interaction

**Fig. 3:**
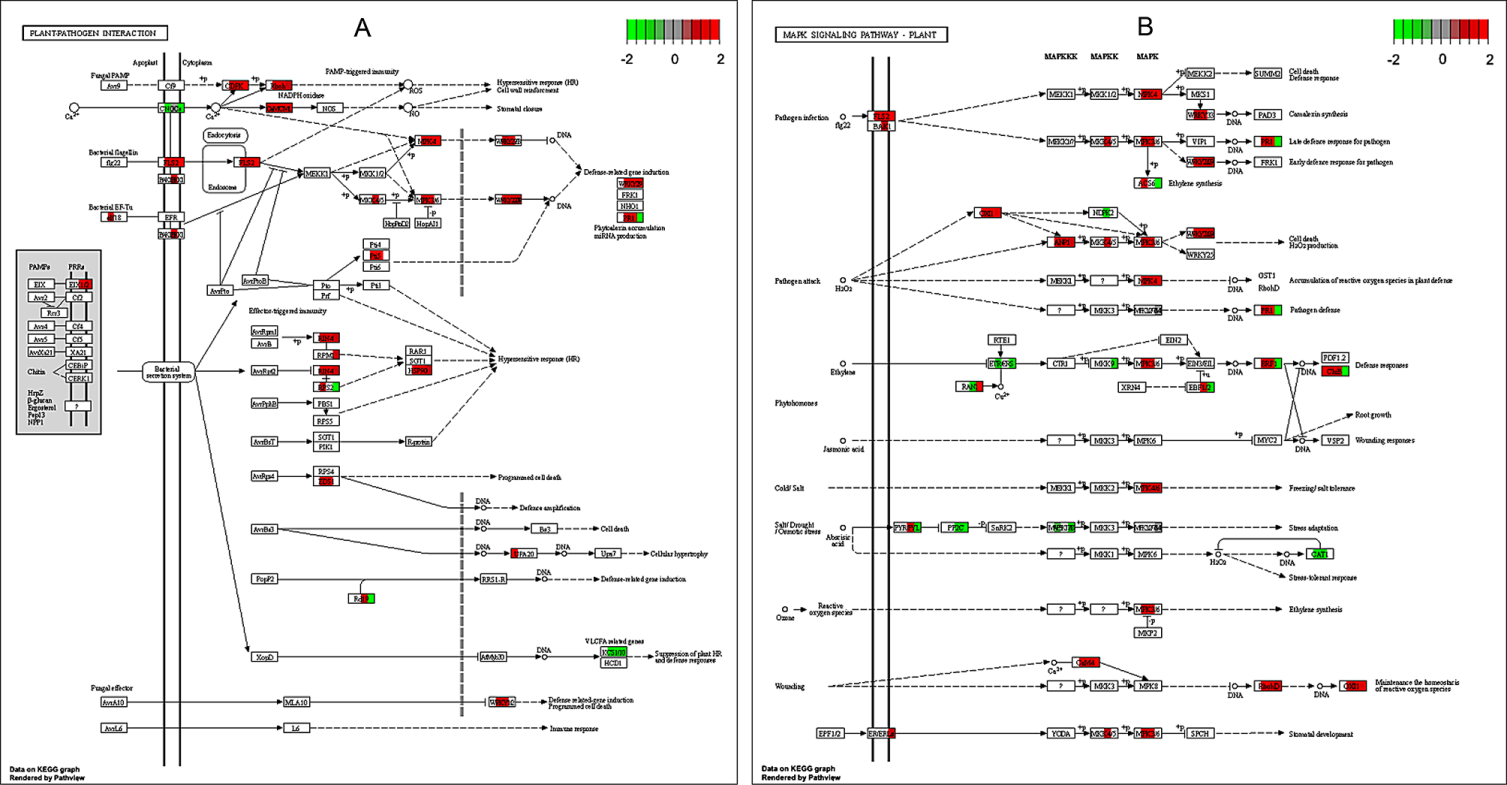
Pathview analysis in ECW50R relative to ECW at each timepoint. (**A**) Plant Pathogen Interaction pathway. (**B**) MAPK Signaling pathway. Each colored node is divided into four parts representing the significant relative gene expression in an order of, between water control, at 1 dpi, 2 dpi and 4 dpi from left to right. Green color represents downregulation, red represents upregulation, grey represents no significant difference, and the white color represents gene expression levels are not influenced by *Xe*-P6 inoculation

It was observed that a significant number of genes involved in the Plant Pathogen Interaction (PPI) pathway exhibited upregulation at 1, 2, and 4 dpi in the ECW50R genotype when compared to ECW (Fig. 3A). Notably, the pathogen-associated molecular pattern (PAMP) LRR receptor-like protein kinases, specifically flagellin sensing 2 (FLS2), showed strong upregulation starting at 1 dpi. Genes interacting with the Ca2 + pathway, such as Calcium-Dependent Protein Kinases (CDPK) and Calmodulins (CaM/CML) and Respiratory Burst Oxidase Homolog (Rboh) protein also displayed upregulation (Fig. 3A). Activation of these receptors is known to lead to the formation of reactive oxygen species (ROS), triggering PAMP-Triggered Immunity (PTI). Additionally, genes central to the PPI pathway, including Mitogen-Activated Protein Kinase 4 (MPK4), WRKY DNA-binding protein 29 (WRKY29) and Pathogenesis-Related Protein 1 (PR1) were upregulated. Upon comparing timepoints within each genotype, it was evident that most defense-related proteins exhibited upregulation in ECW50R as well as downregulation in ECW. A notable difference between these lines was observed in FLS2 and the Ca + 2 dependent pathway, including CDPK and Rboh. RPM1-Interacting Protein 4 (RIN4) was upregulated at all three timepoints in ECW50R; however, in ECW, it was upregulated only at 4 dpi and not differentially expressed at 1 and 2 dpi (Fig. S3 and S4). The list of significantly differentially expressed (absolute log2FC ≥ 1 and p.adj ≤ 0.05) genes annotated as FLS2 in KEGG database along with their respective fold change values in ECW50R relative to ECW at 1-, 2- and 4-days post inoculation are presented in supplementary Table S3.

### Expression of genes involved in MAPK signaling pathway

Alterations in the expression of genes involved in the MAPK (Mitogen-Activated Protein Kinase) Signaling Pathway were observed, particularly in the comparison between ECW50R and ECW at different timepoints. It was noted that several genes associated with MAPK cascades showed upregulation at 1 and 2 dpi in ECW50R as compared to ECW. These included MPK4, MPK3, and WRKY transcription factors WRKy33 and WRKY29 (Fig. 3B). Additionally, 1-aminocyclopropane-1-carboxylic acid synthase 6 (ACS6), responsible for ethylene synthesis, showed upregulation at 1 dpi, followed by downregulation at 4 dpi. The pathway involving H_2_O_2_ production showed significant upregulation, with Oxidative Signal-Inducible 1 (OXI1) being upregulated at 1, 2, and 4 dpi, but not in the water control group. Furthermore, enzymes related to ethylene synthesis, specifically Ethylene Response Factor 1 (ERF1) and Chitinase B (ChiB), exhibited upregulation at 1 and 2 dpi, followed by downregulation at 4 dpi (Fig. 3B). Higher levels of transcripts of genes involved in MAPK signaling pathway in ECW50R compared to ECW is consistent with the hypothesis that ECW50R’s resistance is mediated by greater activation of MAPK signaling pathway.

### Expression of genes involved in plant hormone signal transduction

Differentially expressed genes (DEGs) associated with various plant hormones, including auxin, cytokinin, abscisic acid (ABA), brassinosteroid, ethylene (ET), salicylic acid (SA), and jasmonic acid (JA), were identified within the plant hormone signal transduction pathway. Specifically, genes related to salicylic acid (SA), such as Nonexpresser of Pathogenesis-Related Genes 1 (NPR1), exhibited downregulation at 1, 2, and 4 dpi in ECW50R compared to ECW (Fig. S5). In contrast, the downstream gene Pathogenesis-Related Protein 1 (PR-1) showed upregulation at 1 and 2 dpi, but downregulation at 4 dpi. Similarly, the Jasmonate-ZIM Domain (JAZ) enzyme associated with ubiquitin-mediated proteolysis linked to JA demonstrated upregulation at 1 and 2 dpi and downregulation at 4 dpi (Fig. S5). Ethylene Response Factor 1 (ERF1), associated with ethylene (ET) synthesis, displayed upregulation at 1 and 2 dpi and downregulation at 4 dpi. Moreover, several auxin-related genes were observed to be upregulated at 4 dpi, indicating their potential role in the later stages of the response (Fig. S5).

### In planta population growth of wild type and Δ*fliC* mutant of *X. euvesicatoria*

**Fig. 4:**
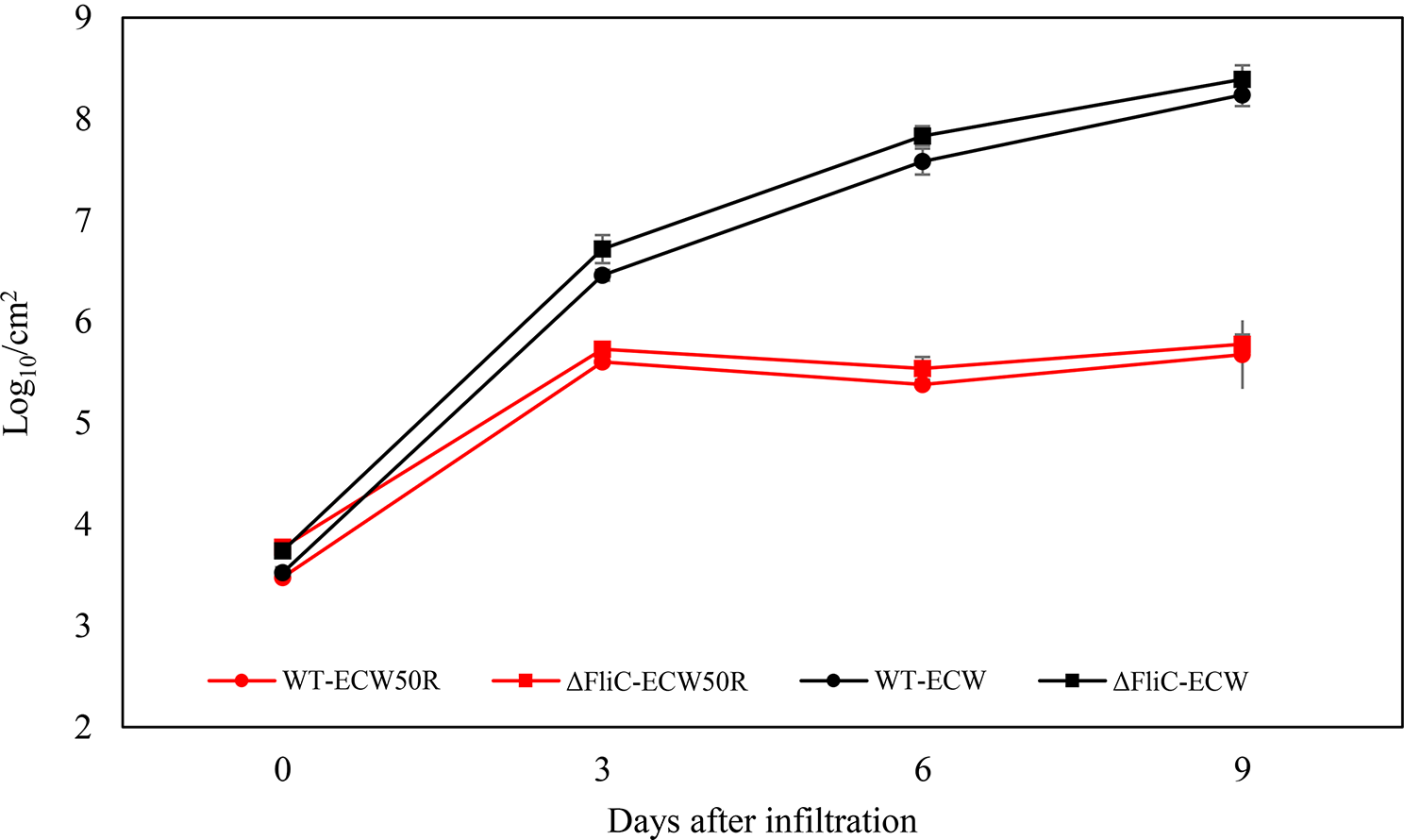
*In planta* populations of *X. euvesicatoria* (21_52_F1) Wild type strain (circle marker) and the corresponding *fliC* deletion mutant (ΔfliC -rectangular marker) in leaves of resistant pepper line ECW50R (red line) and susceptible pepper line ECW (black line) infiltrated with 10^5^ CFU/mL bacterial suspension at 0, 3, 6, and 9 days after infiltration. The error bars indicate standard errors. Paired sample t-test was conducted to determine significant differences

To investigate the resistance mechanism in ECW50R and its potential association with the FLS2-mediated pathway, known for its interaction with the flg22 component of bacterial flagella [41, 42, 45], an *in planta* population growth experiment was carried out. A *fliC* mutant of *Xe* (ΔFliC), specifically engineered to disrupt flagella-related interactions, was created. This mutant strain was then infiltrated into both the ECW and ECW50R lines to assess the effect on bacterial population growth in comparison to the wild-type strain. Surprisingly, our findings revealed no apparent differences between the wild-type and mutant strains concerning their *in planta* growth in both genotypes (Fig. 4).

### Histochemical assay using DAB staining

**Fig. 5:**
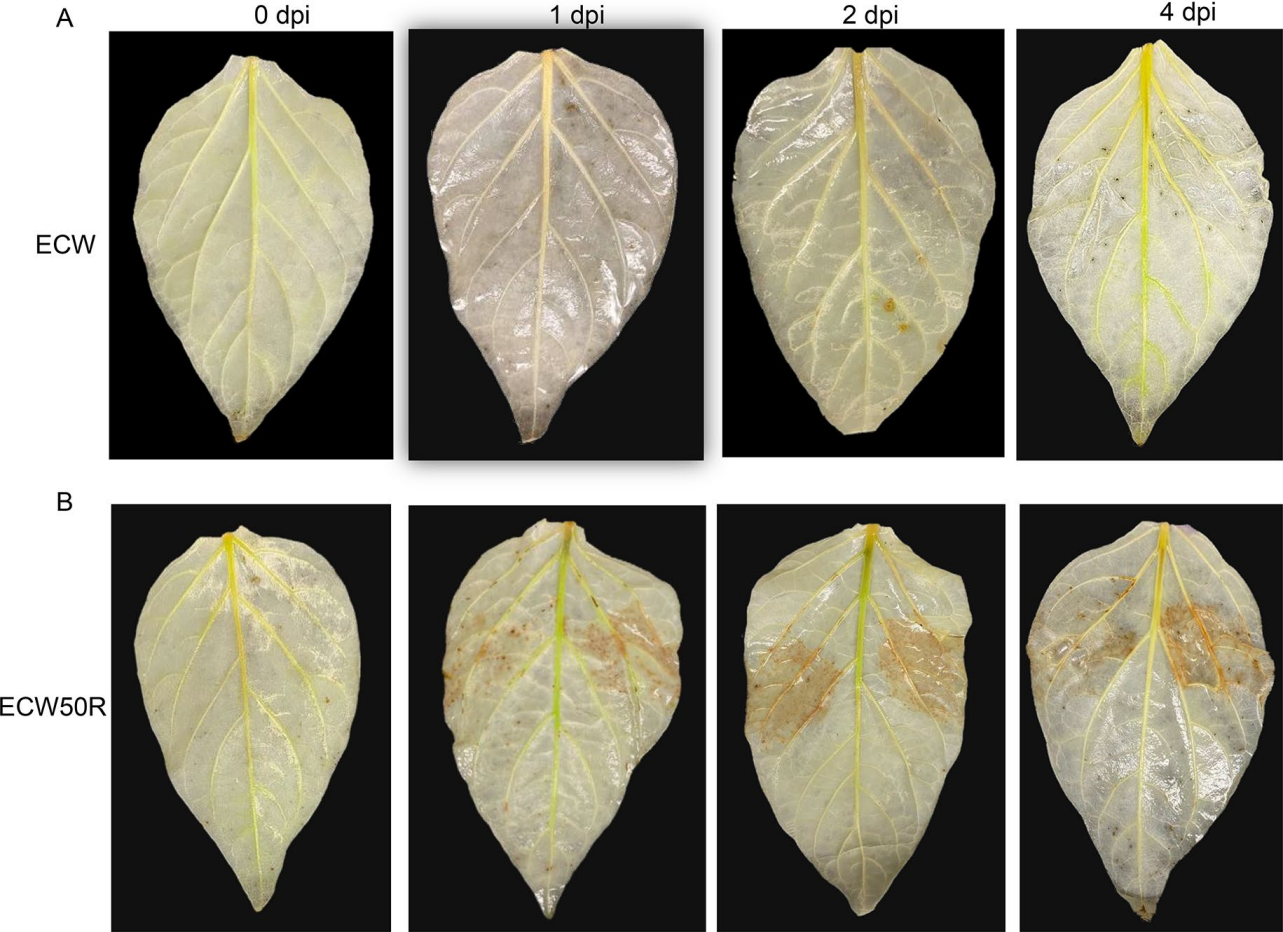
Detection of hydrogen peroxide using DAB staining on leaves of (**A**) ECW and (**B**) ECW50R in response to *X. euvesicatoria* strain 21_52_F1 infiltrated with 10^8^ CFU/mL bacterial suspension at 0, 1, 2 and 4 days after infiltration (dpi)

Given the transcriptomics analyses in Fig. 3 indicating an increase in ROS, we undertook to verify hydrogen peroxide (H_2_O_2_) production and the accumulation of ROS in leaf tissue. Therefore, we conducted DAB staining on ECW50R, and ECW pepper leaves inoculated with *Xe* strain 21_52_F1. Areas subjected to infiltration in ECW50R exhibited a distinct dark brown coloration upon DAB staining at 1, 2, and 4 dpi, indicating the presence of oxidative burst (Fig. 5B). In contrast, no dark brown coloration was observed in ECW at 1, 2, and 4 dpi (Fig. 5A). There was no brown discoloration at 0 dpi in both genotypes, indicating a baseline absence of ROS production at the initial timepoint (Fig. 5). This distinctive pattern between ECW50R and ECW pepper genotypes suggests that the resistance by *bs5* in response to *Xe* infection is accompanied by increased ROS production, typical of PAMP-triggered immunity (PTI).

### Electrolyte Leakage in ECW50R and ECW with P6 Strain

**Fig. 6:**
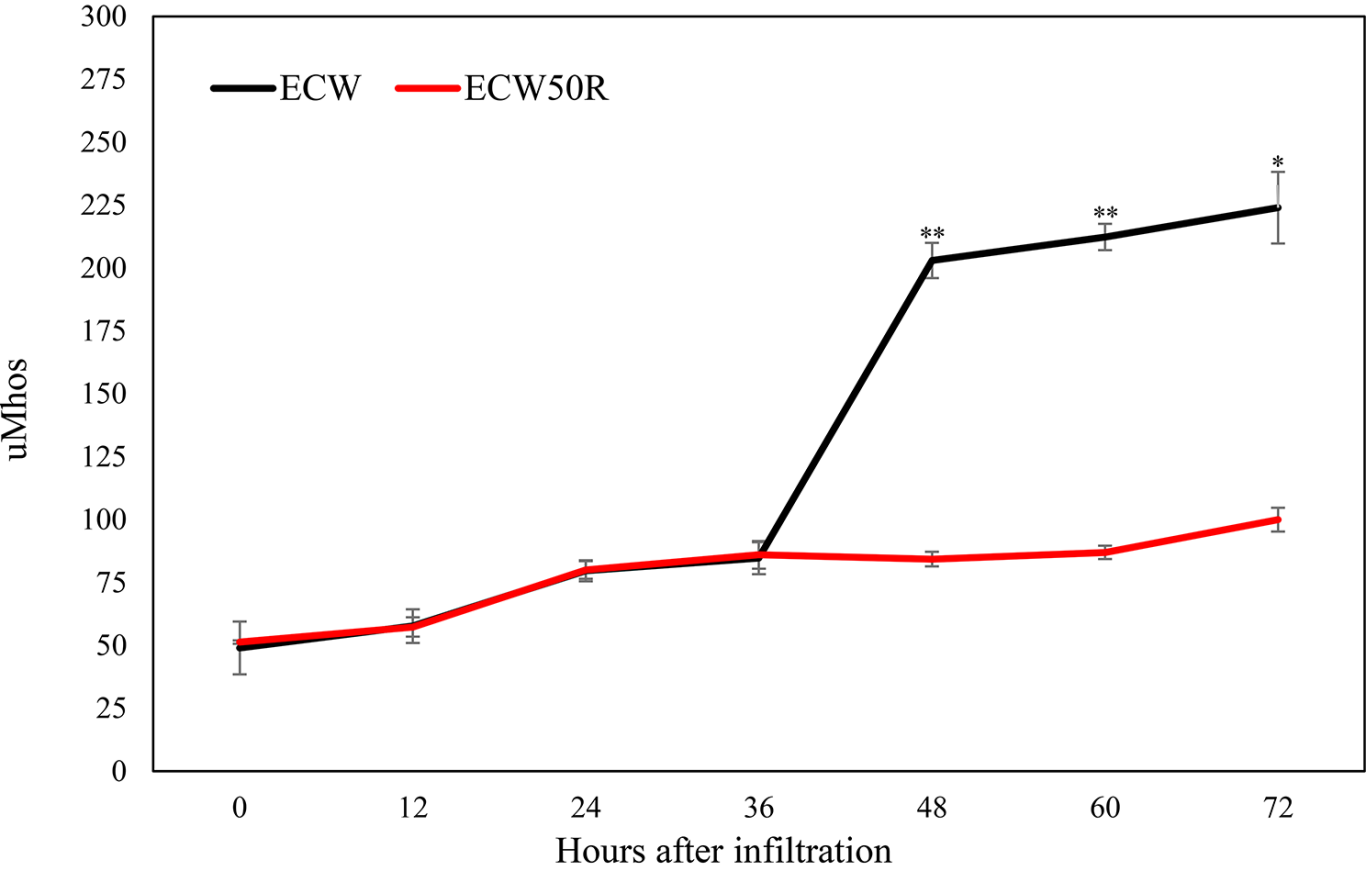
Electrolyte leakage from resistant pepper line ECW50R (red) and susceptible pepper line ECW (black) infiltrated with 10^8^ CFU/mL bacterial suspension of *X. euvesicatoria* strain 21_52_F1 at 0, 12, 24, 36, 48, 60 and 72 h post infiltration. The error bars indicate standard errors. The star above the bars denotes the significantly different from ECW50R (* = *p* < 0.05, ** = *p* < 0.01). Paired sample t-test was conducted to determine significant differences

To assess whether ECW50R induces rapid cell death or maintains cell membrane integrity at the infection site, an electrolyte leakage experiment was conducted. Electrolyte leakage remained consistently low in both ECW and ECW50R leaves for up to 36 h after infiltration with the *Xe* 21_52_F1 strain. After this period, a significant increase in electrolyte leakage was observed in ECW leaves, while ECW50R leaves did not exhibit a similar increase within 72 h (Fig. 6).

### Hypersensitive response reaction in ECW12346 with P3, P4 and P5 strains

**Fig. 7:**
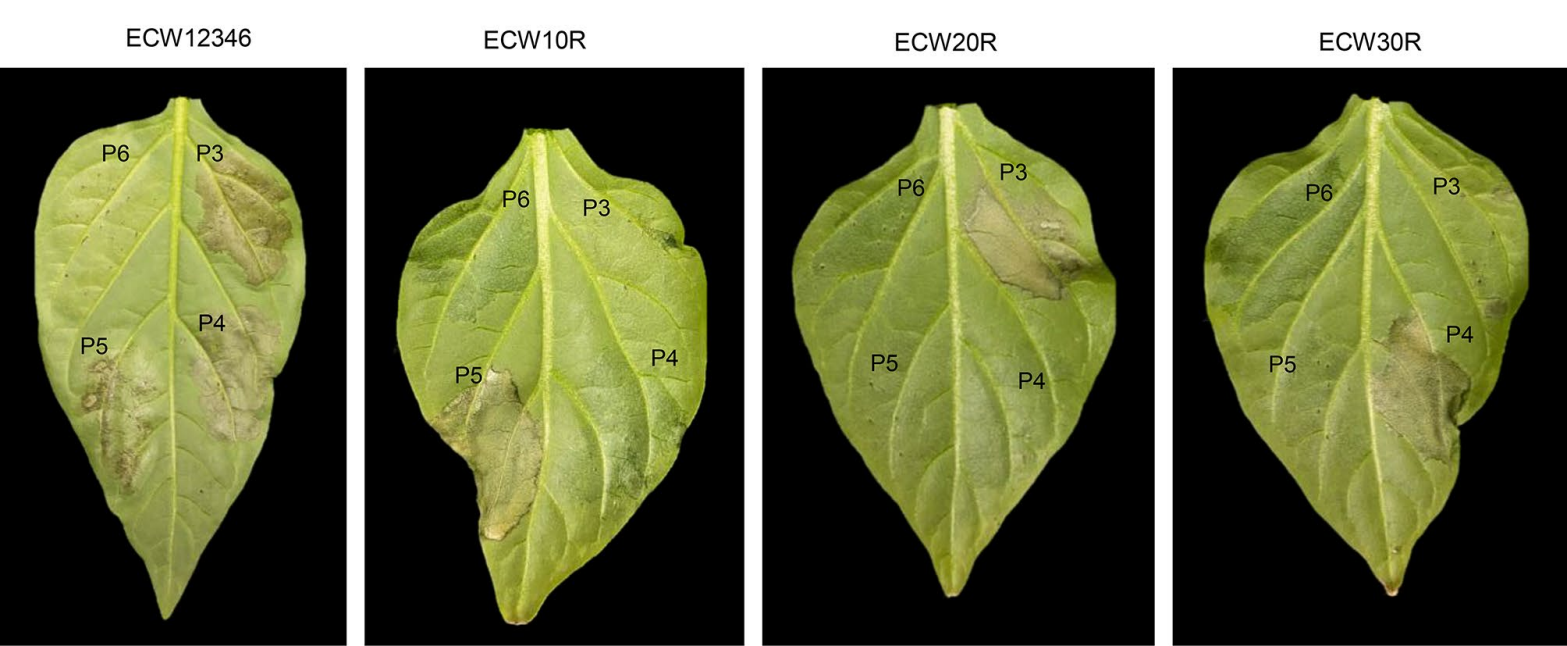
Hypersensitive and susceptible reaction of P3, P4, P5 and P6 in pepper lines ECW12346, ECW10R, ECW20R and ECW30R infiltrated with a bacterial suspension adjusted to 10^8^ CFU/mL. ECW10R, ECW20R, and ECW30R (each carrying *Bs1*,* Bs2*, and *Bs3*, respectively) exhibited individual HR reactions with P5, P3 and P4, respectively. P6, lacking avirulence genes *avrBs1*,* avrBs2*, and *avrBs3*, displayed water-soaked symptoms across all dominant resistance lines. HR was observed in ECW12346, a genotype carrying *Bs1*,* Bs2*,* Bs3*,* bs5*, and *bs6*, when infiltrated with P3, P4, and P5 strains whereas P6 exhibited an incompatible reaction in a non-HR manner. Pictures were taken at 3 days post inoculation (dpi)

Hypersensitive and susceptible reactions were assessed to determine whether the *bs5* gene impairs the effector delivery in host plant and the gene for gene response. A hypersensitive reaction (HR) was observed in ECW12346, a genotype carrying *Bs1*,* Bs2*,* Bs3*,* bs5*, and *bs6*, when infiltrated with P3, P4, and P5 strains, which contained only *avrBs2*,* avrBs3* or *avrBs1*, respectively (Fig. 7). The pepper line, ECW123, containing *Bs1*,* Bs2*, and *Bs3*, also exhibited individual HR reactions with P5, P3 and P4, respectively (Fig. 7). P6, lacking avirulence (*avr*) genes *avrBs1*,* avrBs2*, and *avrBs3*, displayed water-soaked symptoms across all dominant resistance lines after 72 h. However, in ECW12346, no apparent symptoms (no obvious watersoaking) were observed, as previously described [10]. These findings suggest that *bs5* does not impede the effector delivery system, as evidenced by the HRs observed in ECW12346 with a *bs5* background.

## Discussion

In this study, we present a comprehensive transcriptomic analysis designed to elucidate the molecular mechanisms underlying resistance conferred by the recessive resistance gene, *bs5*, to BSP by comparing with the susceptible cultivar ECW. The *bs5* gene, known for its robust resistance across all pathogen races of *Xe*, represents a promising genetic resource for breeding programs seeking durable resistance in pepper cultivars. The comparative analysis between the resistant genotype carrying *bs5* and its susceptible counterpart, *Bs5*, not only reveals transcriptional dynamics governing the host’s response to bacterial infection, but also provides a valuable resource for the strategic development of more efficacious management approaches through resistance breeding strategies.

Map-based cloning of *bs5* revealed that this resistant allele, located in ~ 535 Kbp region on chromosome 3, encodes a CYSTM protein with a 2-amino acid residue shorter length (6 bp of coding DNA) than the wild-type Bs5 protein [17, 18]. CYSTM proteins are believed to be involved in cellular defense mechanism, thereby boosting the plant’s ability to tolerate stress and resist diseases [46]. Szabó et al. [18] argued that mutations in *bs5* might interfere with translocon formation, by inhibiting the crucial apparatus of type III secretion system for delivering effector molecules into plant cells, potentially hindering effector delivery and contributing to the observed resistance in ECW50R plants. However, our observation that infiltrating suspensions of bacterial strains carrying *avrBs1*,* avrBs2*, or *avrBs3* into leaves of differential line ECW12346 carrying *Bs1*,* Bs2*,* Bs3*,* bs5* and *bs6* resulted in HR indicates recognition between avirulence genes and dominant resistant genes (Fig. 7). This demonstrates that *bs5* does not disrupt the effector delivery system.

It is widely acknowledged that plants have developed a variety of resistance proteins, serving to activate immune responses against pathogen infections [7]. Within this repertoire, plants employ cell surface-specific receptor proteins to detect pathogen-secreted effectors, thereby regulating immunity through PTI [7, 47]. PTI is activated by pathogen-associated molecular patterns (PAMPs) and relies on basal defense mechanisms to limit the colonization of invading pathogens. Given its non-race-specificity, PTI is anticipated to confer durable and broad-spectrum resistance [48]. In the context of our study, we observed significant enrichment of pathways and DEGs associated with PTI. It is noteworthy that the observed differential gene expression was more prominent at 2 dpi. This implies that recognition of *Xe* by pepper plants occurs early in the infection process and within a relatively short timepoint.

Significant differences in gene expression between susceptible (ECW) and resistant (ECW50R) lines in response to infection were observed in the plant pathogen interaction (PPI) pathway. Following *Xe* inoculation, a substantial downregulation of genes in PPI pathway was observed in the susceptible ECW cultivar. In sharp contrast, the ECW50R line exhibited a significant upregulation of genes involved in PPI pathway. Flagellin sensing 2 (FLS2) stands out as the most distinctive response, being upregulated in ECW50R and downregulated in ECW. FLS2 is known for its role in recognition of flagellin (flg22 – epitope of bacterial flagellin) thereby triggering a cascade of signaling events that result in a robust defense response and strengthened plant immune system [41, 42, 43]. FLS2 has been shown to act as defense response against many pathogenic bacteria including *Xanthomonas* [49–52]. The significant differential regulation of FLS2 in the two lines led to the hypothesis that the resistance mechanism in ECW50R is primarily mediated by FLS2 recognition of flg22. To test this hypothesis, a mutated *Xe* P6 strain with deletion of *fliC* gene (ΔFliC) lacking the flg22 domain was used. If FLS2 expression is not induced in ECW50R line in the absence of a functional flg22 domain, a significant decrease in defense responses might be expected. Surprisingly, bacterial growth in ECW50R and ECW were similar between the wild-type and mutant strains (Fig. 4), suggesting that the FLS2-mediated pathway may not be the exclusive determinant of resistance in ECW50R. In a previous study, *fliC* mutants of *Pseudomonas syringae pv. tomato* and *E. coli* showed expression profiles of PAMP-induced genes in Arabidopsis similar to flagellin-producing strains, also indicating that flagellin does not uniquely contribute to PAMP-induced transcriptional changes [53]. While bacterial flagellin is a prominent activator of FLS2, it is important to note that the regulation of FLS2 is part of a complex network of signaling pathways and other related factors can also lead to FLS2 activation. For instance, ethylene signaling [41, 54, 55], repression of TOE1/TOE2 [56] or other unidentified factors can all contribute to upregulation of FLS2. Gómez-Gómez and Boller (2000) suggested that FLS2 is a constituent of a preexisting recognition system, in which expression of FLS2 remains unaltered in response to flg22 itself. In the susceptible ECW line, FLS2 was consistently downregulated at 1, 2, and 4 dpi compared to the water control. This downregulation could be a strategic move by the bacteria to evade detection or a result of effector molecules suppressing FLS2 expression. Alternatively, susceptible hosts may prioritize the resources that facilitate pathogen establishment over robust defense mechanisms.

Numerous components associated with Ca2 + signaling, including CDPK, CaM/CML, and Rboh, were found to be upregulated in ECW50R in response to *Xe* infection. Calcium ion (Ca2+) signals are essential for modulating plant defense responses to combat pathogens [57] and are activated in pepper in response to pathogen infections [29, 58, 59]. We found that OXI1 showed upregulation from 1 dpi onwards, leading to increased expression of WRKY factors, such as WRKY22 and WRKY29, in ECW50R compared to ECW. WRKY22 serves as a marker of PTI, when induced by the detection of flg22, and is linked to resistance responses [60]. The upregulation of RbohD and OXI1 genes corresponds to hydrogen peroxide accumulation, resulting in a burst of reactive oxygen species (ROS). Dark brown coloration indicative of hydrogen peroxide accumulation was observed in the infiltrated areas of ECW50R leaves after *Xe* inoculation (Fig. 5B). Therefore, we believe that the observed increase of ROS activity in the ECW50R leaves inoculated with *Xe* can be attributed to the inducible expression of genes associated with defense response pathways. One significant outcome of Ca2 + signaling is the HR, a hallmark of plant innate immunity, particularly in incompatible systems. HR is typically associated with production of ROS, NO, SA, and Ca2 + fluxes [61]. Despite observing ROS production in ECW50R, the quantity of ROS generated may not be sufficient for extensive cell death, making it a form of non-HR resistance. Jones et al. (2002) observed no rapid electrolyte leakage increase in ECW12346 following P6 strain infiltration, but noted a significant rise when leaves were infiltrated with P3 strain, indicating a non-hypersensitive resistance mechanism. Hypersensitive response, typically associated with ETI, involves rapid localized cell death at the infection site [61], with electrolyte leakage measurement serving as a quantifier of cell death. ETI signaling is initiated following direct or indirect recognition of pathogen effectors by NLRs and activation of ETI results in enhanced resistance and HR [62]. The absence of rapid electrolyte leakage in ECW50R and HR in this study provides compelling evidence for the absence of ETI, indicating that the defense response observed in *bs5* background may be primarily associated with PTI.

MAPK cascades are recognized as pivotal players in plant responses to pathogen infections [63]. In context of ECW50R, both MPK3/6 and MPK4, key components of MAPK cascades, were found to be upregulated. MAPK has been shown to enhance rice resistance to *X. oryzae pv. oryzicola* and to withstand drought stress by phosphorylating and activating MPK6 and MPK3 [64]. A considerable array of MAPK genes were also differentially expressed in response to *X. oryzae pv. oryzae* infection in rice, *X. campestris pv. vesicatoria* (*X. euvesicatoria)* infection in pepper and *X. citri subsp. citri* infection in citrus [29, 30, 64]. The upregulation of MAPK components in ECW50R highlights their crucial involvement in defense against *Xe*.

Plant hormones like JA, SA, and ET are crucial for plant defense signaling pathways [65]. Several DEGs involving in these phytohormone signaling pathways were found to be enriched in this study. Notably, the JAZ enzyme, integral to ubiquitin-mediated proteolysis in the JA pathway, and ERF1, linked to ET synthesis, showed upregulation at 1 and 2 dpi but downregulation at 4 dpi. Conversely, NPR1, a regulator associated with SA, was downregulated in ECW50R compared to ECW. Plant hormones exhibit diverse responses to pathogens across various pathosystems, as citrus activates SA, ET, and JA against *X. citri subsp. citri* [30], tomato regulates ET and JA to combat *X. perforans* [26], and pepper with *Bs1* resistance increases ET and SA against *X. euvesicatoria* [29]. In contrast, exclusively JA-signaling genes are upregulated in the *X. axonopodis pv. glycines* system in soybean [66]; citrus downregulates ET and JA against *X. citri subsp. citri* [67]; and peach leaves with *X. arboricola pv. pruni* show no enrichment in SA or JA-signaling genes [68]. These findings suggest a pathosystem-dependent modulation of ET, SA and JA-signaling pathways in response to pathogens.

In conclusion, this study offers valuable insights into the resistance mechanisms of *bs5* in ECW50R when facing *Xe* infection. The innate immune system of the plant harboring *bs5* resistance gene seems to establish a robust line of defense through PTI, involving the upregulation of key elements such as FLS2, Ca-dependent pathways, Rboh, the generation of ROS, and the activation of defense responses. The findings revealed the upregulation of multiple resistance-related signaling pathways, forming a complex and interconnected defense network. Multiple genes identified to be upregulated early upon inoculation of *Xe* in ECW50R could be targets for novel engineering strategies to achieve bacterial spot resistance for other hosts such as tomato and for knockouts to study resistance mechanisms.

### Electronic supplementary material

Below is the link to the electronic supplementary material.

Supplementary Material 1

## Data Availability

No datasets were generated or analysed during the current study.

## Authors and affiliations

Department of Plant Pathology, University of Florida, Gainesville, Florida, USA.

Aastha Subedi, Gerald V. Minsavage, Erica M. Goss, Anuj Sharma, Jeffrey B. Jones.

Southwest Florida Research & Education Center, University of Florida, Immokalee, Florida, USA.

Pamela D. Roberts.

Emerging Pathogens Institute, University of Florida, Gainesville, Florida, USA.

Erica M. Goss.

Department of Horticultural Sciences, University of Florida, Gulf Coast Research and Education Center, Wimauma, Florida, USA.

Anuj Sharma.
