# Supplementary material for: Insights into bs5 resistance mechanisms in pepper against Xanthomonas euvesicatoria through transcriptome profiling
Source: BMC Genomics. 2024 Jul 23;25:711. doi: 10.1186/s12864-024-10604-8 (PMC11267861; doi:10.1186/s12864-024-10604-8)
Supplement: Supplementary file 1 — Supplementary Material 1 [file 12864_2024_10604_MOESM1_ESM.docx]

Table S1: STATISTIC ANALYSIS OF PEPPER CLEAN READS IN 30 LIBRARIES FOR RNA-SEQ

| Sample Name | Total Reads | Total base | Number of genes | Alignment rate (%) | No feature hits | Ambiguous hits | Low Quality hits | Not aligned reads | Not unique reads | BioSample accessions |
| --- | --- | --- | --- | --- | --- | --- | --- | --- | --- | --- |
|  | (R1 + R2) | pairs |  |  |  |  |  |  |  |  |
| ECW_Wa_1 | 44891230 | 6778575730 | 23392 | 66.07 | 2032924 | 6037 | 493296 | 7327358 | 858855 | SAMN40948227 |
| ECW_Wa_2 | 73802078 | 11144113778 | 24061 | 76.06 | 4102284 | 9571 | 1441907 | 8054267 | 1895652 | SAMN40948228 |
| ECW_Wa_3 | 41087158 | 6204160858 | 23170 | 62.64 | 1576243 | 5188 | 418707 | 7411631 | 882731 | SAMN40948229 |
| ECW_0_1 | 28697996 | 4333397396 | 23133 | 94.89 | 1757316 | 4869 | 432709 | 505147 | 874993 | SAMN40948230 |
| ECW_0_2 | 25513596 | 3852552996 | 23941 | 94.98 | 1704492 | 4365 | 420568 | 419701 | 736055 | SAMN40948231 |
| ECW_0_3 | 33081826 | 4995355726 | 23389 | 95.39 | 1971759 | 4496 | 487760 | 506370 | 1035520 | SAMN40948232 |
| ECW_1_1 | 33639528 | 5079568728 | 23382 | 93.97 | 2019603 | 5092 | 509756 | 746483 | 821626 | SAMN40948233 |
| ECW_1_2 | 28289724 | 4271748324 | 23184 | 93.9 | 1694040 | 3608 | 480236 | 609580 | 735015 | SAMN40948234 |
| ECW_1_3 | 33133684 | 5003186284 | 23116 | 93.74 | 1759879 | 5272 | 530580 | 759981 | 843620 | SAMN40948235 |
| ECW_2_1 | 29870958 | 4510514658 | 23258 | 94.58 | 1576792 | 4690 | 431172 | 585032 | 713939 | SAMN40948236 |
| ECW_2_2 | 45041078 | 6801202778 | 23428 | 67.39 | 1611164 | 4948 | 597751 | 7003677 | 988763 | SAMN40948237 |
| ECW_2_3 | 45332678 | 6845234378 | 23257 | 62.4 | 1625012 | 4289 | 486274 | 8235918 | 902577 | SAMN40948238 |
| ECW_4_1 | 41319074 | 6239180174 | 22622 | 61.96 | 1405317 | 4380 | 419195 | 7609112 | 736198 | SAMN40948239 |
| ECW_4_2 | 30670828 | 4631295028 | 22882 | 94.18 | 1417529 | 4855 | 501604 | 632086 | 764375 | SAMN40948240 |
| ECW_4_3 | 33173012 | 5009124812 | 23335 | 93.54 | 1688368 | 5877 | 597035 | 761590 | 728962 | SAMN40948241 |
| ECW50R_Wa_1 | 29868494 | 4510142594 | 23553 | 94.37 | 1857239 | 4929 | 522199 | 565035 | 982223 | SAMN40948242 |
| ECW50R_Wa_2 | 30221132 | 4563390932 | 23288 | 94.23 | 1937720 | 5114 | 582219 | 566156 | 855619 | SAMN40948243 |
| ECW50R_Wa_3 | 31536486 | 4762009386 | 23520 | 94.31 | 2080429 | 5686 | 608075 | 581020 | 791566 | SAMN40948244 |
| ECW50R_0_1 | 25821724 | 3899080324 | 22998 | 94.97 | 1629727 | 4385 | 428823 | 422729 | 834879 | SAMN40948245 |
| ECW50R_0_2 | 29233052 | 4414190852 | 23276 | 94.93 | 1851486 | 5760 | 476139 | 491304 | 870461 | SAMN40948246 |
| ECW50R_0_3 | 44825800 | 6768695800 | 23513 | 66.66 | 1906171 | 5879 | 569513 | 7154629 | 820352 | SAMN40948247 |
| ECW50R_1_1 | 41882690 | 6324286190 | 23090 | 63.46 | 1261821 | 5406 | 470835 | 7378973 | 761394 | SAMN40948248 |
| ECW50R_1_2 | 41270290 | 6231813790 | 23033 | 64.15 | 1417261 | 5136 | 469568 | 7128806 | 715641 | SAMN40948249 |
| ECW50R_1_3 | 28064064 | 4237673664 | 23160 | 94.09 | 1457343 | 5279 | 419409 | 609857 | 759199 | SAMN40948250 |
| ECW50R_2_1 | 25941020 | 3917094020 | 23044 | 93.96 | 1316974 | 4893 | 398962 | 575684 | 610007 | SAMN40948251 |
| ECW50R_2_2 | 29159264 | 4403048864 | 23030 | 93.8 | 1476594 | 5459 | 501740 | 641052 | 768855 | SAMN40948252 |
| ECW50R_2_3 | 30040998 | 4536190698 | 22949 | 94.36 | 1447469 | 5755 | 463532 | 603860 | 775263 | SAMN40948253 |
| ECW50R_4_1 | 30999420 | 4680912420 | 22878 | 92.11 | 1625711 | 5529 | 576223 | 921127 | 789274 | SAMN40948254 |
| ECW50R_4_2 | 27283958 | 4119877658 | 22786 | 92.5 | 1579818 | 4964 | 495600 | 764635 | 612842 | SAMN40948255 |
| ECW50R_4_3 | 26168232 | 3951403032 | 22669 | 93.14 | 1519326 | 4725 | 480911 | 647218 | 593694 | SAMN40948256 |

Table S2: Table showing the stringent differentially expressed genes (DEGs) with an absolute log_2_FC ≥ 8 and p.adj ≤ 0.05 in ECW50R relative to ECW with respect to water control,1-, 2- and 4-days post inoculation. NS represents non-significant and non-highlighted values represent significant under the threshold of log_2_FC ≥ 8.

| Gene-Function | Control | One_dpi | Two_dpi | Four_dpi |
| --- | --- | --- | --- | --- |
| LOC107864472 - geraniol 8-hydroxylase | -11.66 | -11.44 | -11.61 | -11.08 |
| LOC107864473 - geraniol 8-hydroxylase | -10.50 | -10.63 | -10.91 | -9.98 |
| LOC107864308 - chaperone protein DnaJ-like | -10.46 | -10.15 | -10.27 | -9.58 |
| LOC107869262 - Unknown | -7.69 | -10.05 | -10.47 | -11.40 |
| LOC107864264 - sm-like protein LSM5 | -5.99 | -9.81 | -8.72 | NS |
| LOC107866432 - 2-hydroxyisoflavanone dehydratase | -9.87 | -9.48 | -9.91 | NS |
| LOC107873682 - Uncharacterized | -9.96 | -9.39 | -9.82 | -10.13 |
| LOC107875179 - Unknown | -8.35 | -9.35 | -9.59 | NS |
| LOC107873118 - Uncharacterized | -8.42 | -8.77 | NS | NS |
| LOC107872614 - acetyltransferase At3g50280-like | NS | -8.55 | -5.97 | NS |
| LOC107866492 - Unknown | -8.16 | -8.07 | -8.09 | NS |
| LOC107868150 - Uncharacterized | NS | -7.80 | -8.69 | -7.54 |
| LOC107868022 - Uncharacterized | NS | -7.39 | -8.70 | NS |
| LOC107873036 - Unknown | NS | -7.27 | -7.48 | -8.22 |
| LOC107873049 - alanine--tRNA ligase | NS | -7.25 | -8.71 | NS |
| LOC107868192 - Uncharacterized | NS | -7.23 | -7.94 | -8.28 |
| LOC107872313 - Uncharacterized | NS | -6.69 | -5.67 | -8.36 |
| LOC107867554 - probable cellulose synthase A catalytic subunit 6 [UDP-forming] | NS | -4.45 | -8.35 | NS |
| LOC107862611 - Uncharacterized | NS | -4.33 | -10.60 | -5.90 |
| LOC107867716 - LRR receptor-like serine/threonine-protein kinase RGI1 | NS | -3.98 | -8.38 | NS |
| LOC107872613 - Uncharacterized | NS | -3.43 | NS | -8.29 |
| LOC107854253 - WAT1-related protein At4g30420 | NS | -2.68 | NS | -8.65 |
| LOC107866602 - Unknown | NS | 4.57 | 3.91 | 8.80 |
| LOC107879815 - Unknown | 9.67 | 8.05 | 8.94 | 8.08 |
| LOC107867771 - Uncharacterized | 7.76 | 8.50 | 8.53 | 8.07 |
| LOC107877565 - heat shock 70 kDa protein | NS | NS | -10.04 | -12.91 |
| LOC107843460 - non-specific lipid-transfer protein 2 | NS | NS | -10.36 | -11.19 |
| LOC107862779 - miraculin | NS | NS | NS | -10.96 |
| LOC107858813 - carbonic anhydrase, chloroplastic | NS | NS | NS | -10.79 |
| LOC107852837 - GDSL esterase/lipase 6 | NS | NS | NS | -10.63 |
| LOC107854042 - defensin-like protein | NS | NS | -4.00 | -10.19 |
| LOC107862771 - proteinase inhibitor type-2 | NS | NS | NS | -9.65 |
| LOC107859881 - gibberellin-regulated protein 11 | NS | NS | NS | -8.94 |
| LOC107845932 - Unknown | NS | NS | NS | -8.87 |
| LOC107857372 - alcohol acyl transferase 1 | NS | NS | NS | -8.86 |
| LOC107854939 - UDP-glycosyltransferase 74G1 | NS | NS | NS | -8.84 |
| LOC107853492 - laccase-14 | NS | NS | NS | -8.45 |
| LOC107878728 - indole-3-acetic acid-amido synthetase GH3.6 | NS | NS | NS | -8.43 |
| LOC107840150 - squalene monooxygenase SE1 | NS | NS | NS | -8.42 |
| LOC107877425 - Unknown | NS | NS | NS | -8.32 |
| LOC107878318 - endochitinase 4 | NS | NS | NS | -8.31 |
| LOC107841134 - probable aspartic proteinase GIP2 | NS | NS | -4.00 | -8.27 |
| LOC107856520 - Uncharacterized | -7.17 | NS | NS | -8.27 |
| LOC107874840 - EG45-like domain containing protein | NS | NS | NS | -8.26 |
| LOC107867711 - alcohol dehydrogenase 1 | NS | NS | NS | -8.25 |
| LOC107855209 - aquaporin TIP1-1 | NS | NS | NS | -8.23 |
| LOC107866027 - kunitz trypsin inhibitor 5 | NS | NS | NS | -8.11 |
| LOC107865651 - ethylene-responsive proteinase inhibitor 1 | NS | NS | NS | -8.11 |
| LOC107854358 - very-long-chain aldehyde decarbonylase CER1-like | NS | NS | NS | -8.09 |
| LOC107854790 - putative disease resistance protein At3g14460 | NS | NS | 9.79 | 7.14 |
| LOC107854440 - Uncharacterized | 6.49 | NS | NS | 8.01 |
| LOC107873879 - bidirectional sugar transporter SWEET12 | NS | NS | NS | 8.06 |
| LOC107847469 - disease resistance protein RGA2 | NS | NS | 9.23 | 8.26 |
| LOC107852707 - 14 kDa proline-rich protein DC2.15 | NS | NS | NS | 11.25 |
| LOC107847470 - Uncharacterized | NS | NS | 21.81 | 21.84 |
| LOC107855648 - Uncharacterized | NS | NS | 25.82 | 26.58 |
| LOC107877989 - auxin-binding protein ABP19a | NS | NS | -11.99 | NS |
| LOC107845879 - protein TAP1 | NS | NS | -9.00 | NS |
| LOC107853990 - cyclin-A2-2 | 8.01 | NS | NS | NS |

Table S3: List of significantly expressed (absolute log2FC ≥ 1 and p.adj ≤ 0.05) genes annotated as FLS2 in KEGG database along with their respective fold change values in ECW50R relative to ECW at 1-, 2- and 4-days post inoculation. NS represents non-significant and non-highlighted values represent significant under the threshold of log_2_FC ≥ 1.

| FLS2_loci | One_dpi | Two_dpi | Four_dpi |
| --- | --- | --- | --- |
| LOC107877942 | -1.79 | -0.88 | 0.92 |
| LOC107859246 | NS | 1.94 | 1.13 |
| LOC107877593 | 1.39 | 2.25 | 1.15 |
| LOC107856496 | 1.67 | 1.47 | 1.18 |
| LOC107868249 | 2.95 | 1.82 | 1.65 |
| LOC107870159 | 1.19 | 1.49 | 1.70 |
| LOC107868254 | 2.06 | 2.59 | 1.71 |
| LOC107870158 | NS | 1.04 | 2.00 |
| LOC107859251 | 2.19 | 2.66 | 2.03 |
| LOC107859245 | NS | NS | 3.38 |
| LOC107868265 | 1.59 | 2.22 | 3.50 |
| LOC107855770 | NS | NS | 3.52 |
| LOC107868412 | 1.41 | 2.68 | 3.66 |
| LOC107854613 | NS | NS | 3.81 |
| LOC107869344 | NS | NS | 3.91 |
| LOC107871528 | NS | NS | 5.27 |
| LOC107859253 | -1.60 | -1.51 | NS |
| LOC107870169 | NS | 1.29 | NS |
| LOC107868209 | 1.27 | 1.33 | NS |
| LOC107855783 | NS | 2.09 | NS |
| LOC107868347 | 3.15 | 3.12 | NS |
| LOC107868390 | 2.71 | 3.32 | NS |
| LOC107868391 | 3.35 | 5.13 | NS |
| LOC107840981 | -1.03 | NS | NS |
| LOC107868296 | 1.03 | NS | NS |
| LOC107868374 | 1.53 | NS | NS |

Figure S1. Significantly enriched (Differentially Expressed - DE, Upregulated-Up and Downregulated-Down) KEGG pathways in ECW50R alone at different timepoints with respect to water control.

Figure S2. Significantly enriched (Differentially Expressed- DE, Upregulated-Up and Downregulated-Down) KEGG pathways in ECW alone at different timepoints with respect to water.


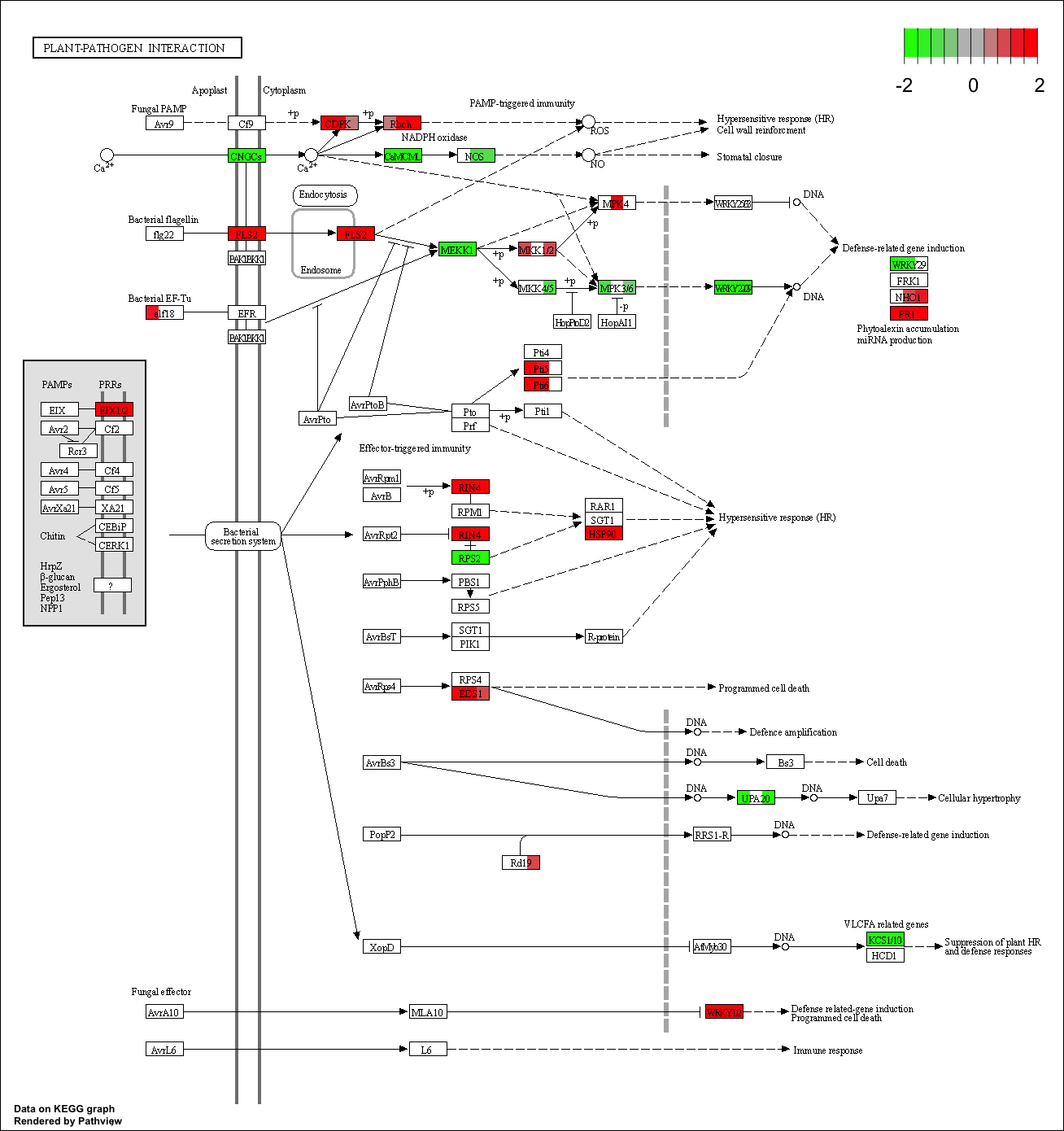


Figure S3. Pathview analysis in plant pathogen interaction pathway in ECW50R only relative to water control at each timepoint. Each colored node is divided into three parts representing the significant relative gene expression in an order at 1 dpi, 2 dpi and 4 dpi from left to right. Green color represents downregulation, red represents upregulation, grey represents no significant difference, and the white color represents gene expression levels are not influenced by *Xe*-P6 inoculation.


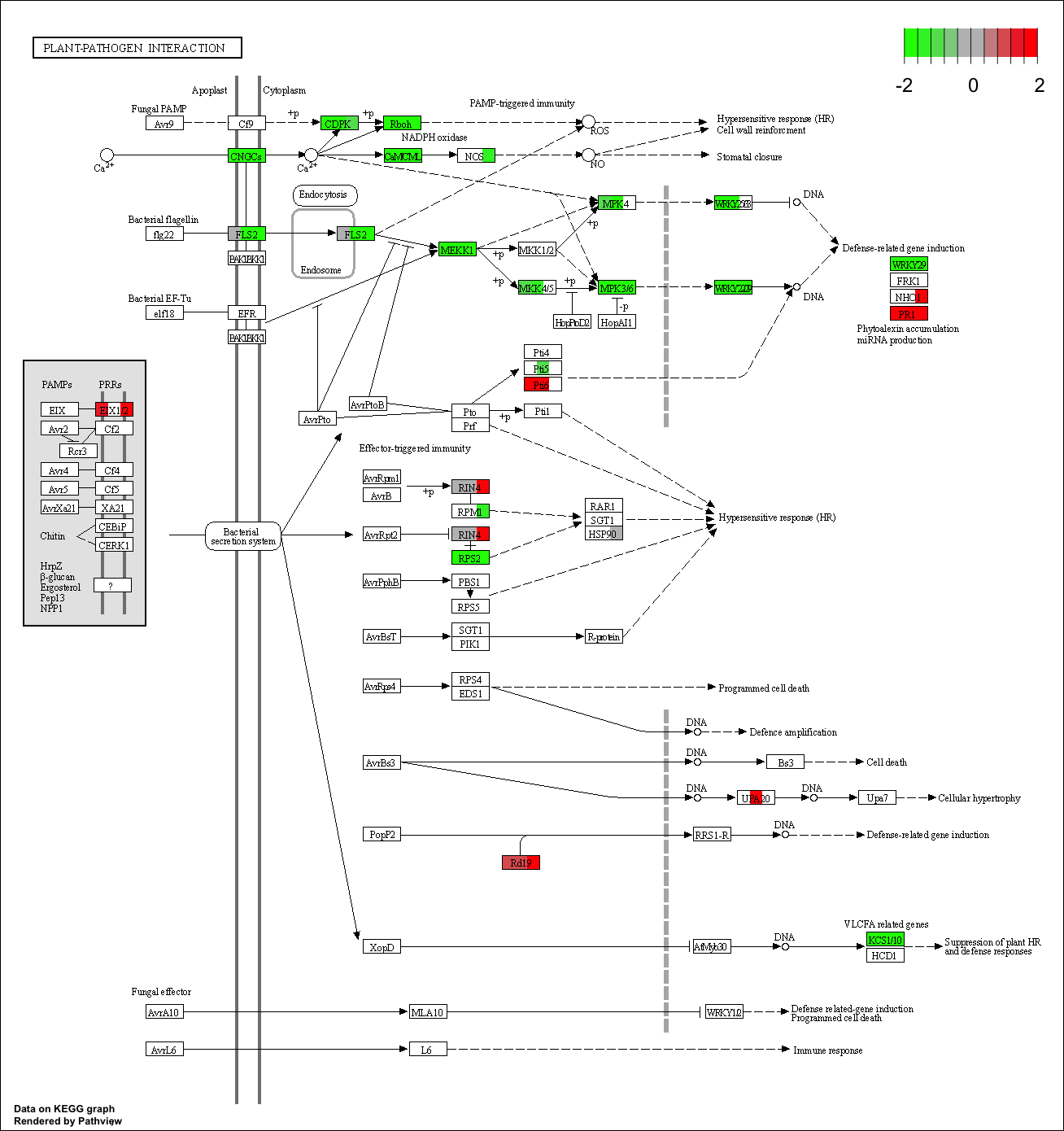


Figure S4. Pathview analysis in plant pathogen interaction pathway in ECW only relative to water control at each timepoint. Each colored node is divided into three parts representing the significant relative gene expression in an order at 1 dpi, 2 dpi and 4 dpi from left to right. Green color represents downregulation, red represents upregulation, grey represents no significant difference, and the white color represents gene expression levels are not influenced by Xe-P6 inoculation.

*
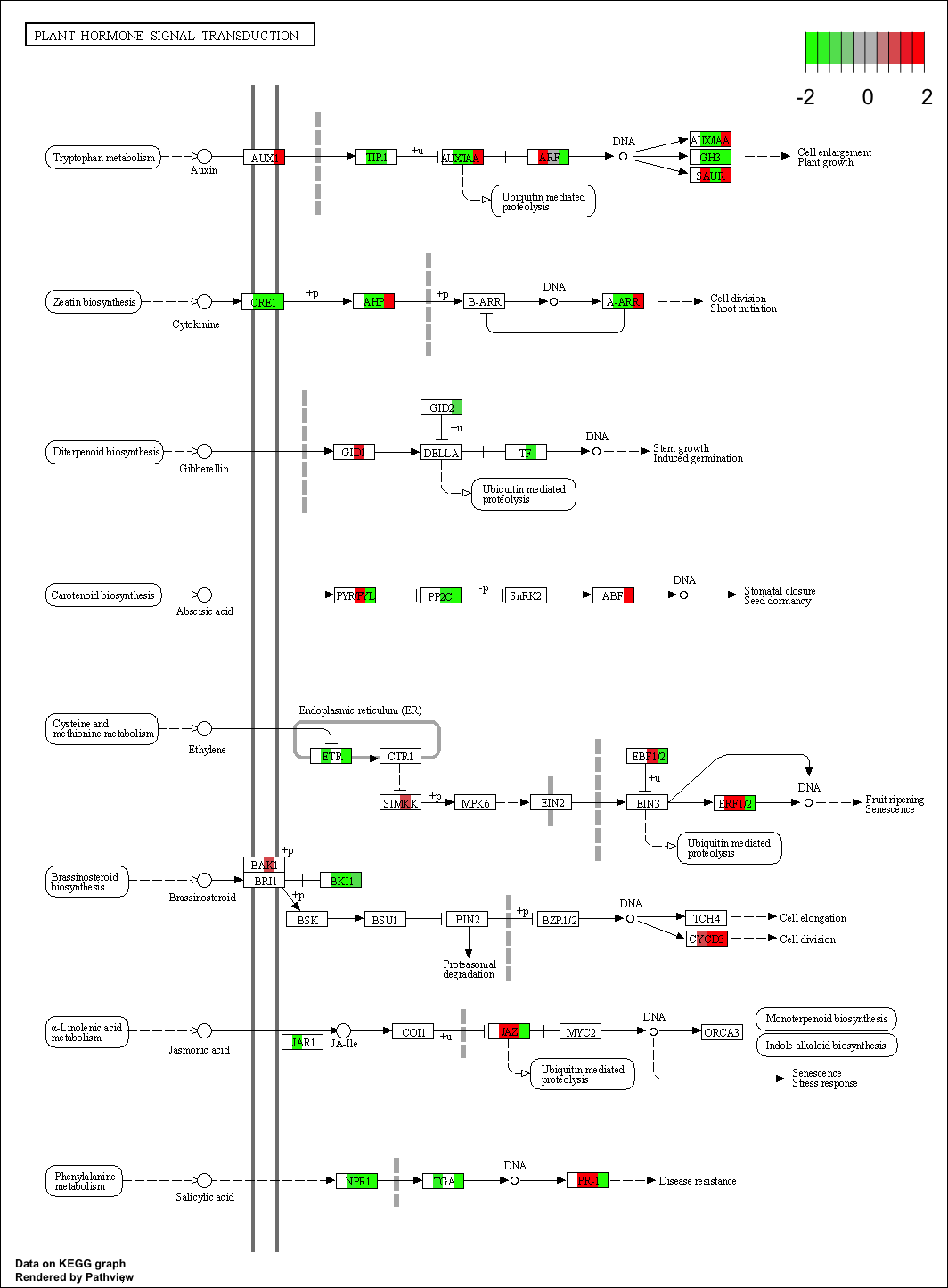
*

Figure S5. Pathview analysis in plant hormone signal transduction pathway in ECW50R relative to ECW at each timepoint. Each colored node is divided into four parts representing the significant relative gene expression in an order of, between water control, at 1 dpi, 2 dpi and 4 dpi from left to right. Green color represents downregulation, red represents upregulation, grey represents no significant difference, and the white color represents gene expression levels are not influenced by *Xe*-P6 inoculation.
